# Supplementary material for: Echocardiographic parameters according to insulin dose in young patients affected by type 1 diabetes
Source: PLoS One. 2020 Dec 28;15(12):e0244483. doi: 10.1371/journal.pone.0244483 (PMC7769446; doi:10.1371/journal.pone.0244483)
Supplement: Attachment — Submitted filename: Answers.docx [file pone.0244483.s001.docx]

PONE-D-20-20811
Echocardiographic parameters according to insulin dose in young patients affected by type 1 diabetes.
PLOS ONE

Please include the following items when submitting your revised manuscript:

- A rebuttal letter that responds to each point raised by the academic editor and reviewer(s). You should upload this letter as a separate file labeled 'Response to Reviewers'.
- A marked-up copy of your manuscript that highlights changes made to the original version. You should upload this as a separate file labeled 'Revised Manuscript with Track Changes'.
- An unmarked version of your revised paper without tracked changes. You should upload this as a separate file labeled 'Manuscript'.

If you would like to make changes to your financial disclosure, please include your updated statement in your cover letter. Guidelines for resubmitting your figure files are available below the reviewer comments at the end of this letter.

A) The financial disclosure was not changed. No figures.

If applicable, we recommend that you deposit your laboratory protocols in protocols.io to enhance the reproducibility of your results. Protocols.io assigns your protocol its own identifier (DOI) so that it can be cited independently in the future. For instructions see: [http://journals.plos.org/plosone/s/submission-guidelines#loc-laboratory-protocols](http://journals.plos.org/plosone/s/submission-guidelines#loc-laboratory-protocols)

When submitting your revision, we need you to address these additional requirements.

1. Please ensure that your manuscript meets PLOS ONE's style requirements, including those for file naming. The PLOS ONE style templates can be found at

https://journals.plos.org/plosone/s/file?id=wjVg/PLOSOne_formatting_sample_main_body.pdf and

https://journals.plos.org/plosone/s/file?id=ba62/PLOSOne_formatting_sample_title_authors_affiliations.pdf

 A) Done following the style requirements.

2.Thank you for including your ethics statement:  "The study was approved by the local ethic committee, and the informed consent was obtained".

Please amend your current ethics statement to include the full name of the ethics committee/institutional review board(s) that approved your specific study.

A) done

Once you have amended this/these statement(s) in the Methods section of the manuscript, please add the same text to the “Ethics Statement” field of the submission form (via “Edit Submission”).

 A) done

3. You indicated that you had ethical approval for your study. In your Methods section, please ensure you have also stated whether you obtained consent from parents or guardians of the minors included in the study

 A) no minors were included in the study.

4. We note you have included a table to which you do not refer in the text of your manuscript. Please ensure that you refer to Table 4 in your text; if accepted, production will need this reference to link the reader to the Table.

A) Thanks. We report the refer of table 4 in the text.

Reviewers' comments:

Reviewer's Responses to Questions

**Comments to the Author**

1. Is the manuscript technically sound, and do the data support the conclusions?

The manuscript must describe a technically sound piece of scientific research with data that supports the conclusions. Experiments must have been conducted rigorously, with appropriate controls, replication, and sample sizes. The conclusions must be drawn appropriately based on the data presented.

Reviewer #1: Yes

Reviewer #2: Yes

2. Has the statistical analysis been performed appropriately and rigorously?

Reviewer #1: Yes

Reviewer #2: Yes

3. Have the authors made all data underlying the findings in their manuscript fully available?

The [PLOS Data policy](http://www.plosone.org/static/policies.action#sharing) requires authors to make all data underlying the findings described in their manuscript fully available without restriction, with rare exception (please refer to the Data Availability Statement in the manuscript PDF file). The data should be provided as part of the manuscript or its supporting information, or deposited to a public repository. For example, in addition to summary statistics, the data points behind means, medians and variance measures should be available. If there are restrictions on publicly sharing data—e.g. participant privacy or use of data from a third party—those must be specified.

Reviewer #1: Yes

Reviewer #2: Yes

4. Is the manuscript presented in an intelligible fashion and written in standard English?

PLOS ONE does not copyedit accepted manuscripts, so the language in submitted articles must be clear, correct, and unambiguous. Any typographical or grammatical errors should be corrected at revision, so please note any specific errors here.

Reviewer #1: Yes

Reviewer #2: Yes

5. Review Comments to the Author

Please use the space provided to explain your answers to the questions above. You may also include additional comments for the author, including concerns about dual publication, research ethics, or publication ethics. (Please upload your review as an attachment if it exceeds 20,000 characters)

Reviewer #1: This is an interesting study to evaluate the association between diastolic myocardial relaxation (IVRT) and insulin-dose. The authors observed positive and significant association between insulin dose and IVRT even after adjusting some clinical factors. I have the several comments to the authors.

Thanks for your comments.

1. Why the authors focus on IVRT? Did the authors see any associations between other diastolic parameters and insulin-dose? Some diastolic parameters may be significant after adjusting for confounders.

A) No linear correlations were detected between IVRT and diastolic parameters. IVRT was significantly correlated to insulin dose. We add a sentence in the results section. Insulin dose was not an independent predictor of E/A, E/e’ or LAVI.

2. Table 4: In the multivariable model, how did the authors select the variables? AIC or BIC based? Stepwise selection? High-dose group has higher heart rate that affects IVRT. I have a concern about the authors detected the difference of heart rate (HR) between high-dose and low-dose groups. The association between IVRT and insulin-dose significant further adjusted for HR?

A) In the statistical section we specified how the variables were chosen. The model was a forced entry model. Thanks for your important suggestion about heart rate. We included heart rate in the model and the IVRT maintained its significance. We report this new model, that includes heart rate, in the revised manuscript and the results were changed accordingly.

Reviewer #2: Thank you for allowing me to review this paper titled “Echocardiographic parameters according to insulin dose in young patients affected by type 1 diabetes”.

Thanks for your comments.

This is a cross-sectional study to assess the association between insulin dose and active diastolic myocardial relaxation.

I recommend the manuscript for publications and have a few comments for revision to share:

The aim is to assess the association between cardiac function insulin dose in “young patients” with type 1 diabetes, but there is no mention of age in the abstract.
In the method section, in addition to age, the median insulin dose (u/kg) needs to be provided.

A) We added mean age of patients in the abstract section and mean age plus median insulin dose in the methods section.

Introduction. I think it will be helpful as well as important to categorize the evidence on cardiovascular events in type 1 vs. since the data is controversial.

A) We changed the paragraph of the introduction and added two more references. We hope that the controversy is more evident.

Method: Despite citing reference 4, some background about the setting, date range, and patient population will be helpful.
I suggest being more specific about: “Clinical and laboratory data were collected”.

A) We added more information in the method section.

Results:

Instead of “worse glycemic control,” I suggest using HbA1C “above target”.

A) we change the expression.

Was there any patient on a closed-loop system?

A) 16 subjects were on pump.

Was there any difference in insulin use based on the type of treatment (MDI vs. pump) even with the current sample size?

A) thanks for this question. We compared the insulin dose between patients on pump and MDI, patients on pump had a lower insulin dose. We add the results in the result section.

Can you please provide some info on why the authors decided to use the median to categorize the insulin usage?

A) We use the median value because there is not a recognized cutoff level of insulin dose. In the multivariable model we use insulin dose as a continuous variable.

Table 3. Does using the variables included in table 4 in the multivariate regression models lead to bias for “unmeasured” variables? Would it be possible to include Table 1 variables in the model?

A) We add a sentence in the limitation of the study to acknowledge the possible effect of unmeasured variables.

Discussion: strength: the authors have described the limitations of the study since this analysis indicates a correlation and not a cause-effect relationship.

A) thanks for the comment.

6. PLOS authors have the option to publish the peer review history of their article ([what does this mean?](https://journals.plos.org/plosone/s/editorial-and-peer-review-process#loc-peer-review-history)). If published, this will include your full peer review and any attached files.


If you choose “no”, your identity will remain anonymous but your review may still be made public.


**Do you want your identity to be public for this peer review?** For information about this choice, including consent withdrawal, please see our [Privacy Policy](https://www.plos.org/privacy-policy).

Reviewer #1: No

Reviewer #2: No

[NOTE: If reviewer comments were submitted as an attachment file, they will be attached to this email and accessible via the submission site. Please log into your account, locate the manuscript record, and check for the action link "View Attachments". If this link does not appear, there are no attachment files.]

While revising your submission, please upload your figure files to the Preflight Analysis and Conversion Engine (PACE) digital diagnostic tool, <https://pacev2.apexcovantage.com/>. PACE helps ensure that figures meet PLOS requirements. To use PACE, you must first register as a user. Registration is free. Then, login and navigate to the UPLOAD tab, where you will find detailed instructions on how to use the tool. If you encounter any issues or have any questions when using PACE, please email PLOS at [figures@plos.org](mailto:figures@plos.org). Please note that Supporting Information files do not need this step.
